# Enhanced PIEZO1 function contributes to the pathogenesis of sickle cell disease

**DOI:** 10.1073/pnas.2514863122

**Published:** 2025-10-02

**Authors:** Luis O. Romero, Manisha Bade, Laila Elsherif, Jada D. Williams, Xiangmei Kong, Adebowale Adebiyi, Kenneth I. Ataga, Shang Ma, Julio F. Cordero-Morales, Valeria Vásquez

**Affiliations:** ^a^Department of Biochemistry and Molecular Biology, Center for Membrane Biology, McGovern Medical School, University of Texas Health Science Center at Houston, Houston, TX 77030; ^b^MD Anderson Cancer Center and University of Texas Health Science Center Graduate School of Biomedical Sciences, Houston, TX 77030; ^c^Division of Hematology, Center for Sickle Cell Disease, University of Tennessee Health Sciences Center, Memphis, TN 38163; ^d^Department of Physiology, University of Tennessee Health Science Center, Memphis, TN 38163; ^e^Children’s Medical Center Research Institute, Department of Pediatrics, University of Texas Southwestern Medical Center, Dallas, TX 75390; ^f^Department of Medical Pharmacology and Physiology, University of Missouri, Columbia, MO 65212; ^g^Department of Anesthesiology and Perioperative Medicine, University of Missouri, Columbia, MO 65212; ^h^NextGen Precision Health, University of Missouri, Columbia, MO 65211

**Keywords:** sickle cell disease, PIEZO1, hemolysis, patch clamp electrophysiology, eicosapentaenoic acid (EPA)

## Abstract

Sickle cell disease (SCD), an inherited blood disorder caused by a mutation in the β-globin gene, is characterized by sickle erythrocytes that are prone to hemolysis, leading to anemia and vaso-occlusion crises. In sickle erythrocytes, hemoglobin aggregation is followed by altered cation permeability and subsequent dehydration. Interventions that restore cation permeability can decrease hemolysis and ameliorate the symptoms associated with SCD. PIEZO1 is a nonselective mechanosensitive cation channel that regulates erythrocyte volume. Gain-of-function (GOF) mutations in PIEZO1 cause hemolytic anemia by increasing cation permeability, leading to erythrocyte dehydration in humans and mice. Although PIEZO1 plays a key role in erythrocyte homeostasis, its role in SCD remains unknown. Here, we demonstrate that the function of the PIEZO1 channel is upregulated in sickle erythrocytes of humans and mice, and this enhancement can be restored through a dietary intervention. We found that PIEZO1 activity in sickle erythrocytes resembles that of the GOF mutation causing hemolytic anemia. A diet enriched in the *ω*-3 fatty acid eicosapentaenoic (EPA) acid decreases PIEZO1 activity in sickle erythrocytes, attenuates hemolysis, and reduces hypoxia-induced sickling. Furthermore, EPA reduces inflammatory markers. We propose that PIEZO1 contributes to the increase in nonselective cationic conductance (i.e., Psickle), which leads to dehydration downstream of hemoglobin polymerization. Our results suggest that reducing PIEZO1 function represents a promising therapeutic strategy to reestablishing normal cation permeability in SCD.

## Significance

Sickle cell disease (SCD) is a life-limiting genetic disorder marked by red blood cell dehydration, hemolysis, and inflammation. The mechanosensitive ion channel PIEZO1 regulates red blood cell volume, but its role in SCD has remained unclear. We show that PIEZO1 function is abnormally elevated in sickle erythrocytes in both humans and mice, resembling gain-of-function mutations that cause hemolytic anemia. Remarkably, a diet enriched in the ω-3 fatty acid eicosapentaenoic acid (EPA) normalizes PIEZO1 activity, reduces hemolysis and hypoxia-induced sickling, and lowers inflammation in a mouse model of SCD. These findings uncover PIEZO1 as a key contributor to SCD pathophysiology and highlight a potential dietary strategy to restore erythrocyte homeostasis.

Sickle cell disease (SCD) is an inherited condition characterized by a mutation in the gene that codes for the hemoglobin subunit β (i.e., sickle hemoglobin, HbS), causing erythrocytes to adopt a sickle-like shape (1). This lifelong and debilitating illness disproportionately impacts Black or African American populations, with the Centers for Disease Control and Prevention reporting an incidence of 1 in 365 births within this demographic (2). Hematopoietic stem cell transplantation, and potentially gene therapy and gene editing approaches, offer curative treatments for SCD (3). Beyond the technical complications (e.g., well-matched donors, age, cost), these options are inaccessible to most patients. Current therapeutic strategies aim to alleviate symptoms and modify the severity of the disease by decreasing hemolytic anemia and vaso-occlusive complications with approved drug therapies (hydroxyurea, L-glutamine, and Crizanlizumab) as well as red blood cell transfusions (1, 3). Despite the availability of newer therapeutic options, the vast majority of individuals with SCD, especially those residing in sub-Saharan Africa, have very limited access to these treatments (4).

Hemoglobin polymerization results in the formation of rigid, fiber-like structures that underlie the pathogenesis of SCD (5, 6). Sickling results in less deformable and more adhesive erythrocytes, resulting in hemolytic anemia, hypoxemia, red blood cell dehydration, and blockage of blood flow. These can cause vaso-occlusive pain crises, organ damage, and endothelial dysfunction (1, 4, 7, 8). Biomarkers of constant hemolysis include increased plasma hemoglobin and indirect bilirubin levels, as well as the presence of inflammatory markers (e.g., cytokines and chemokines) (9–11). The probability of sickle hemoglobin polymerizing is directly proportional to a decrease in the intracellular oxygen concentration and exponentially proportional to the intracellular sickle hemoglobin concentration (12). Interestingly, hemoglobin polymerization alters erythrocyte membrane organization (e.g., loss of membrane asymmetry) and ion permeability (1, 13–15). Several membrane proteins are dysfunctional in this context (e.g., K^+^-Cl^−^ cotransporters, Na^+^-K^+^ pump, ion channels) (16, 17) and are linked to a nonspecific cationic conductance, activated downstream of deoxygenation, termed Psickle (18–21). Psickle leads to dehydration and a subsequent rise in intracellular hemoglobin concentration. This pathophysiological shift is critical in the erythrocyte sickling process, which leads to hemolysis. Identifying the ion channels that contribute to Psickle is crucial for developing new therapeutic strategies.

PIEZO1 is a mechanosensitive, nonselective cation channel that regulates erythrocyte volume (22–25). Lack of PIEZO1 leads to overhydrated erythrocytes and eliminates mechanically induced calcium influx, as demonstrated by calcium imaging experiments (24). Whereas gain-of-function (GOF) point mutations in the *PIEZO1* gene underlie human hereditary xerocytosis, in which the mutant channel increases cation permeability, leading to dehydrated erythrocytes (22, 25–28). A mouse model of a *Piezo1* GOF mutation causing dehydrated xerocytosis mirrors several pathological features observed in SCD, including reduced osmotic fragility and anemia (25). Sickle erythrocytes exhibit reduced intracellular K^+^ and elevated intracellular Ca^2+^ (29, 30)_._ Mechanical stress activates PIEZO1 channels, causing an increase in intracellular Ca^2+^ levels, which in turn opens the Ca^2+^-activated potassium channel (i.e., Gárdos channel) (24). Whether PIEZO1 is a potential target for regulating Ca^2+^ homeostasis in sickle erythrocytes remains to be determined.

Patients with SCD have abnormal erythrocyte membranes characterized by elevated ω-6 fatty acid levels and reduced ω-3 fatty acids (31–34). We have previously established that PIEZO1 function is modulated by the lipid composition of the plasma membrane and that it can be fine-tuned by enriching specific dietary fatty acids in vitro (35). Additionally, we have shown that PIEZO2—a homolog of PIEZO1—can be modulated in vivo by diets enriched in polyunsaturated fatty acids, alleviating symptoms in two unrelated neurogenetic disorders (36, 37). Given the common features of dehydration and increased cation permeability associated with SCD and PIEZO1 GOF mutations, as well as the fatty acid imbalance in SCD blood cell membranes, we aim to determine whether PIEZO1 channel function is altered in SCD.

Here, we demonstrate that PIEZO1 function is enhanced in sickle erythrocytes from human adults and mice with SCD in a sex-independent manner. Notably, we restored normal PIEZO1 function by feeding a mouse model of SCD with a diet enriched in eicosapentaenoic acid (EPA). This dietary intervention decreased plasma hemoglobin, hypoxia-induced sickling, and indirect bilirubin levels and reduced various inflammatory markers. Our findings suggest that PIEZO1 is a key contributor to the altered cation permeability observed in SCD and emphasize the therapeutic potential of targeting this channel to alleviate symptoms associated with SCD and other hematological disorders.

## Results

### Humans with SCD Display Increased PIEZO1 Currents.

**Fig. 1.**
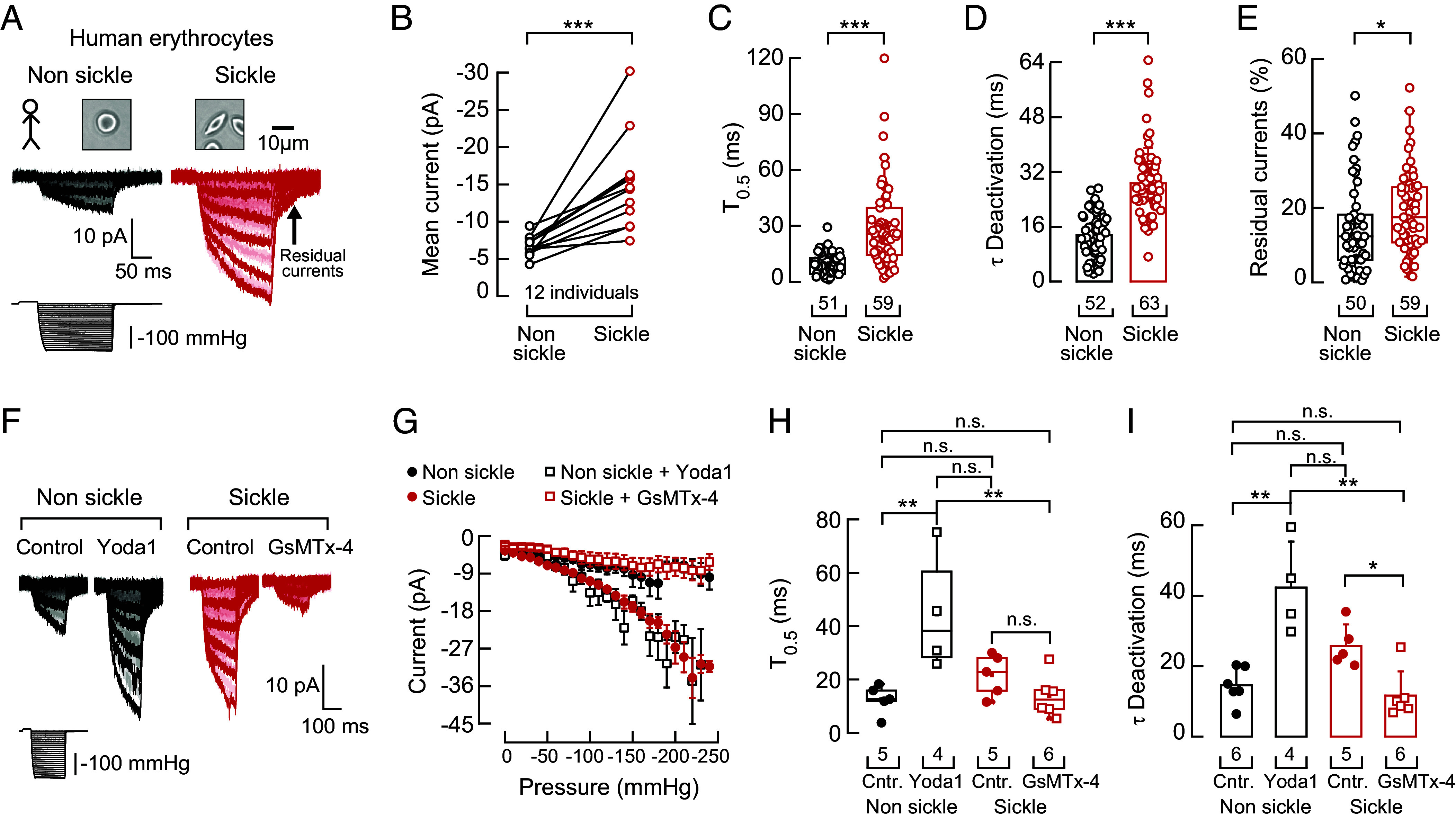
Increased mechanoactivated currents in sickle erythrocytes from SCD patients. (*A*, *Top*) Representative bright field micrographs of sickle and nonsickle erythrocytes from a human with SCD. (*Bottom*) Representative inside-out recordings elicited by negative pressure square pulses at a constant voltage of −60 mV from sickle and nonsickle erythrocytes. (*B*) Mean mechanocurrents elicited by −130 mmHg from nonsickle and sickle erythrocytes. Currents are paired per individual. Two-tailed Wilcoxon matched-pairs signed-ranks test (*P* = 0.0005). (*C*) Time required to reach half of the mechanocurrents maximal value (T_0.5_) elicited by −130 mmHg from nonsickle and sickle erythrocytes. Two-tailed Mann–Whitney test (U = 410, *P* = 2.71^−12^). (*D*) Time constant of deactivation (τ) elicited by maximum negative pressure from nonsickle and sickle erythrocytes. Two-tailed Unpaired *t* test (*t* = 8.97, *P* = 7.43^−15^). (*E*) Percentage of peak current 30 ms after the −130-mmHg stimulus ends from nonsickle and sickle erythrocytes. Two-tailed Mann–Whitney test (U = 1,100, *P* = 0.023). (*F*) Representative inside-out recording elicited by negative pressure square pulses at a constant voltage of −60 mV of control dimethyl sulfoxide (DMSO) or Yoda1 (30 µM)-exposed nonsickle and control or GsMTx-4 (7 µM)-exposed sickle erythrocytes. (*G*) Current–pressure relationships (elicited at −60 mV) of control (DMSO) or Yoda1-exposed nonsickle and control or GsMTx-4 exposed sickle erythrocytes. Symbols are mean ± SEM. (*H*) Time required to reach half of the mechanocurrents maximal value (T_0.5_) elicited by −130 mmHg from control (DMSO) or Yoda1-exposed nonsickle and control or GsMTx-4 exposed sickle erythrocytes. Kruskal–Wallis (H = 9.79, *P* = 0.02) with Dunn’s multiple comparison test. (*I*) Time constant of deactivation (τ) elicited by the maximum negative pressure from control (DMSO) or Yoda1-exposed nonsickle and control or GsMTx-4 exposed sickle erythrocytes. Kruskal–Wallis (H = 14.06, *P* = 0.0028) with Dunn’s multiple comparison test. Bars are mean ± SD. Boxplots show the mean, median, and 75^th^ to 25^th^ percentiles. n is denoted above the *x*-axis. Asterisks indicate values significantly different from the control (*P < 0.05, **P < 0.01, and ***P < 0.001) and n.s. indicates not significantly different. Post hoc *P*-values and source data are available at figshare (40).

To measure PIEZO1 activity in humans with SCD, we collected blood from 12 adult donors during routine clinic visits and patch-clamped their erythrocytes in the inside-out configuration. Individuals with SCD have a varying range (4 to 44%) of sickle erythrocytes in their blood (38, 39). This feature allowed us to measure PIEZO1 activity in sickle and nonsickle erythrocytes under isogenic conditions (Fig. 1*A* and *SI Appendix*, Fig. S1*A*). For all SCD donors, regardless of sex, PIEZO1 mechanocurrents were larger in sickle erythrocytes than in nonsickle ones at every pressure pulse and holding potential (Fig. 1 *A* and *B* and *SI Appendix*, Figs. S1 *B*–*F* and S2*A*). Although −60 mV is not the erythrocyte’s resting potential, we performed most recordings at this holding potential because it maximized separation between erythrocytes under our experimental conditions (*SI Appendix*, Fig. S1*F*). Our results demonstrate that PIEZO1 function is increased in sickle erythrocytes.

We further quantified PIEZO1 gating behavior to identify the salient functional differences between sickle and nonsickle erythrocytes. In sickle erythrocytes, PIEZO1 takes longer to reach half-maximal activation and deactivation (Fig. 1 *C* and *D*). We also observed higher residual currents after ending the mechanical stimuli (Fig. 1 *A* and *E* and *SI Appendix*, Fig. S2*B*). We did not detect differences in the latency of response—the time between the onset of mechanical stimulation and the initiation of PIEZO1 mechanocurrents—between sickle and nonsickle erythrocytes (*SI Appendix*, Fig. S2*C*). Likewise, the linear current–voltage relationships and reversal potentials in both erythrocyte types are similar (*SI Appendix*, Fig. S2*D*). These results indicate that the primary changes in PIEZO1 function in sickle erythrocytes occur in current amplitude, deactivation, and residual current.

We leveraged the available pharmacology of the PIEZO1 channel to further characterize its function in human erythrocytes. Specifically, we challenged membranes of nonsickle erythrocytes with Yoda1, a selective positive modulator of PIEZO1 activity (41), while treating sickle erythrocytes with GsMTx-4, a nonspecific inhibitor of mechanosensitive ion channels (42). Importantly, Yoda1 elicited large mechanocurrents in nonsickle erythrocytes, comparable to those observed in sickle erythrocytes at every pressure step (Fig. 1 *F* and *G* and *SI Appendix*, Fig. S2*E*). Yoda1 also slowed the time to reach half-maximal activation and deactivation and increased the residual currents, similar to those measured in control (DMSO) sickle erythrocytes (Fig. 1 *H* and *I* and *SI Appendix*, Fig. S2 *F* and *G*). On the other hand, GsMTx-4 diminished mechanocurrents and half-maximal activation, while also accelerating deactivation to levels comparable to those observed in nonsickled cells (Fig. 1 *F*–*I*). Neither compound affected the latency of current onset to mechanical stimulation (*SI Appendix*, Fig. S2*H*). Taken together, our functional and pharmacological characterization demonstrates that the PIEZO1 channel mediates an increased cationic permeability in human sickle erythrocytes.

### PIEZO1 Function in Sickle Erythrocytes Resembles a PIEZO1 GOF Mutation.

**Fig. 2.**
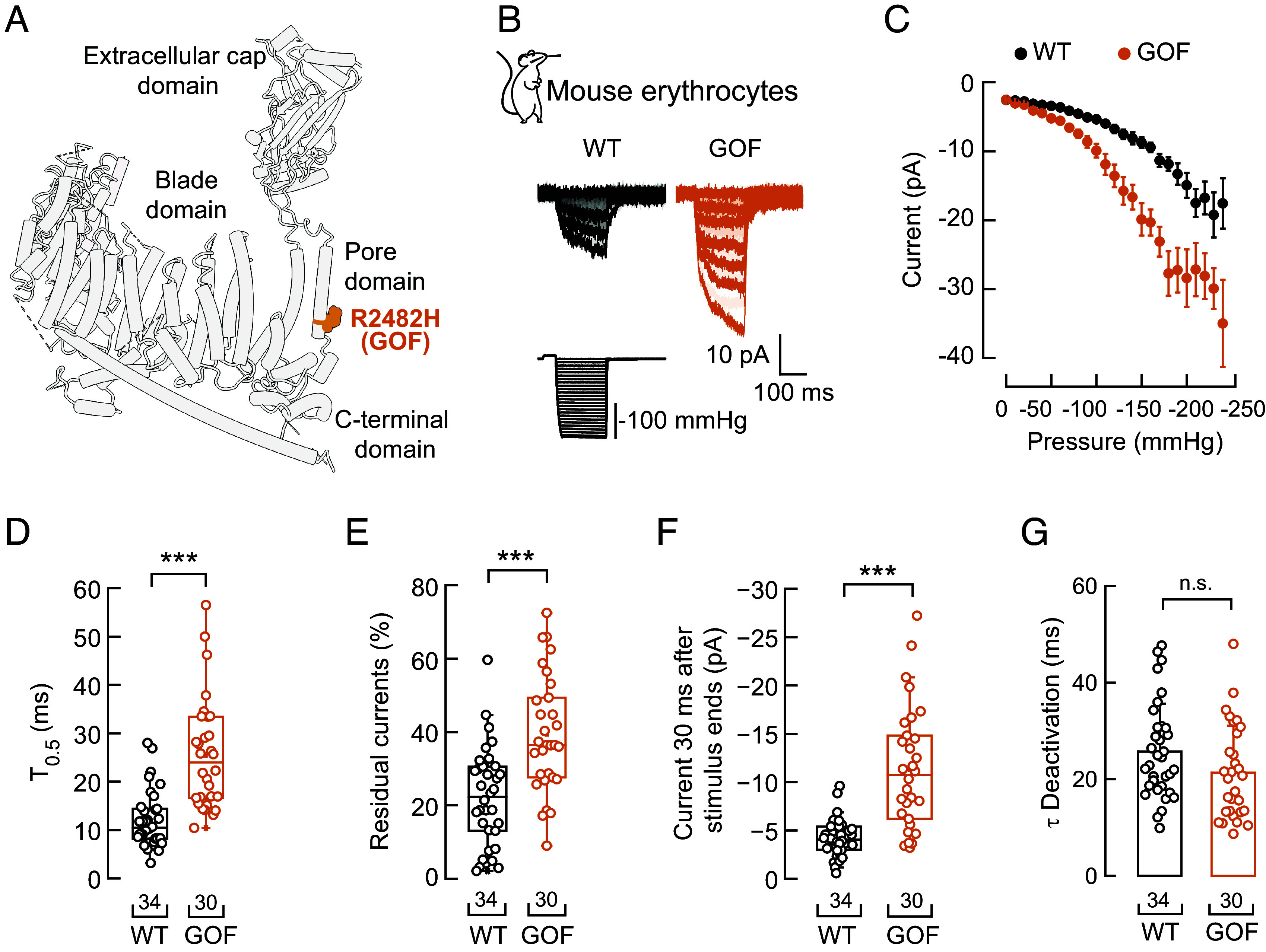
Electrophysiological characterization of the PIEZO1 GOF mutation R2482H in mouse erythrocytes. (*A*) Cylinder representation of the mouse PIEZO1 monomer (PDB ID: 5Z10) highlighting an equivalent residue that causes xerocytosis when mutated (R2482H; GOF) in human *PIEZO1*. (*B*) Representative inside-out recordings elicited by negative pressure square pulses at a constant voltage of −60 mV from WT and PIEZO1 GOF mouse erythrocytes. (*C*) Current–pressure relationships (elicited at −60 mV) from WT (n = 34) and PIEZO1 GOF (n = 30) mouse erythrocytes. Symbols are mean ± SEM. (*D*) Time required to reach half of the mechanocurrents maximal value (T_0.5_) elicited by −130 mmHg from WT and PIEZO1 GOF mouse erythrocytes. Two-tailed Unpaired *t* test (*t* = 120, *P* = 1.53^−8^). (*E*) Percentage of peak current 30 ms after the −130-mmHg stimulus ends from WT and PIEZO1 GOF mice erythrocytes. Two-tailed Unpaired *t* test (t = 4.7, *P* = 1.48^−5^). (*F*) Current remaining 30 ms after the −130-mmHg stimulus ends from WT and PIEZO1 GOF mice erythrocytes. Two-tailed Unpaired *t* test (t = 6.22, *P* = 4.77^−8^). (*G*) Time constant of deactivation (τ) elicited by maximum negative pressure from WT and PIEZO1 GOF mouse erythrocytes. Two-tailed Unpaired *t* test (*t* = 1.78, *P* = 0.08). Boxplots show the mean, median, and 75^th^ to 25^th^ percentiles. n is denoted above the *x*-axis. Asterisks indicate values significantly different from the control (***P < 0.001). n.s. indicates not significantly different. Post hoc *P*-values and source data are available at figshare (40).

The functional features of PIEZO1 in human sickle erythrocytes are reminiscent of the human PIEZO1 GOF mutations that cause xerocytosis, previously characterized when transfected in mammalian cell lines (43). To directly measure the currents of PIEZO1 GOF in erythrocytes, we performed patch-clamp experiments from a hematopoietic lineage-specific mouse line expressing the GOF *Piezo1 R2482H* (25) (Fig. 2*A*) and its corresponding littermates. Following mechanical stimulation, the GOF channel exhibited larger current amplitudes, slower activation kinetics, and larger residual currents than WT (Fig. 2 *B*–*F*). On the other hand, we did not find differences in the τ of deactivation (Fig. 2*G*). The enhanced mechanosensitivity and persistent currents observed in erythrocytes expressing the *R2482H* mutation resemble the functional features we measured for PIEZO1 in sickle human erythrocytes (Fig. 1). Parenthetically, we carried out a pharmacological characterization of PIEZO1 currents in mouse erythrocytes. Consistent with our results from humans, we found that Yoda1 increased PIEZO1 mechanocurrents, and GsMTx-4 reduced the currents in wild-type (WT) mouse erythrocytes (*SI Appendix*, Fig. S3). Together, we characterize PIEZO1 mechanocurrents at the macroscopic level in human and mouse erythrocytes.

### Elevated PIEZO1 Currents in a Mouse Model of SCD.

**Fig. 3.**
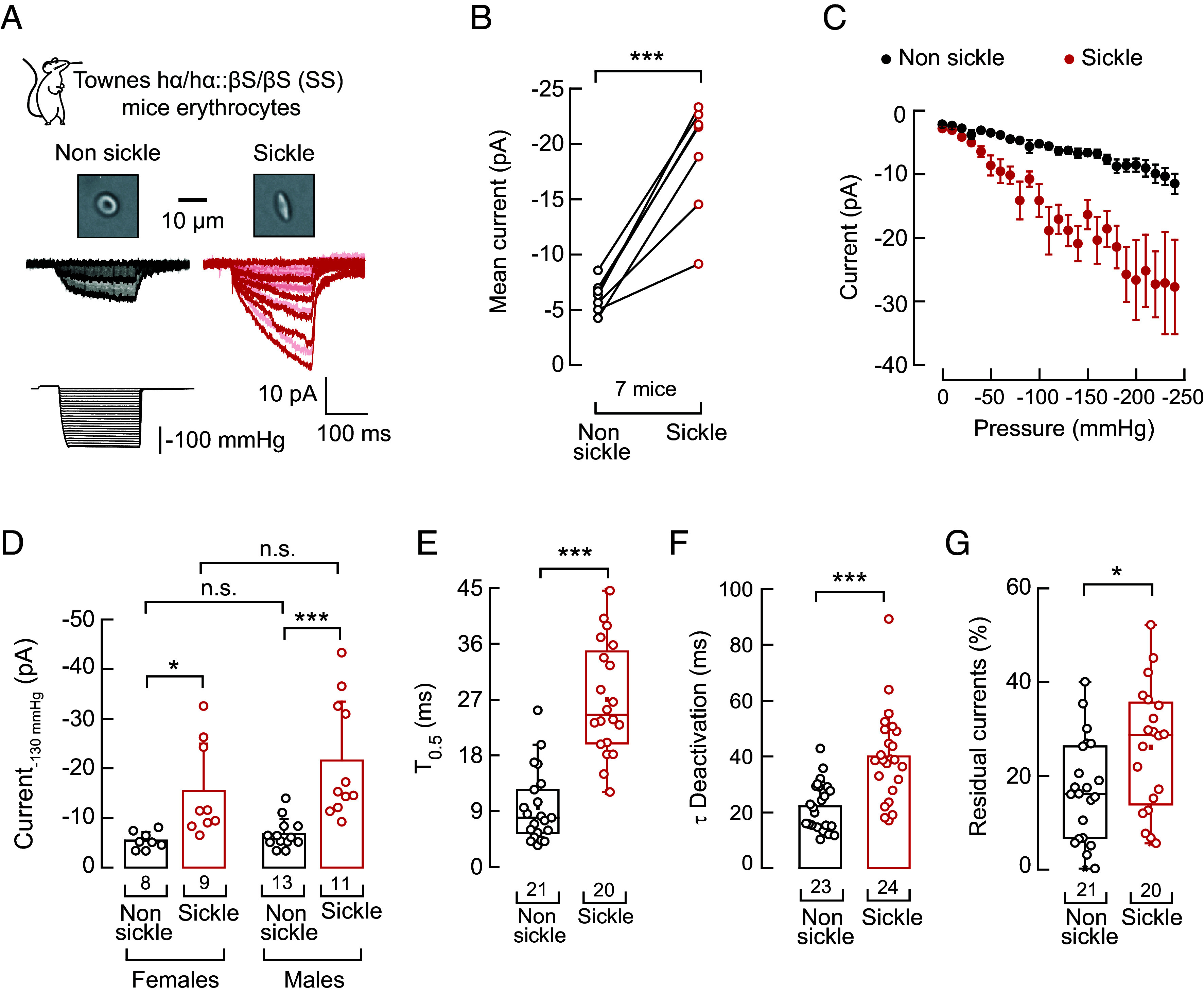
Enhanced PIEZO1 function in sickle erythrocytes from Townes SCD mice. (*A*, *Top*) Representative bright field micrographs of nonsickle and sickle erythrocytes from the Townes mouse model of SCD. (*Bottom*) Representative inside-out recordings elicited by negative pressure square pulses at a constant voltage of −60 mV from nonsickle and sickle erythrocytes. (*B*) Mean mechanocurrents elicited by −130-mmHg from nonsickle and sickle erythrocytes. Currents are paired per mouse. Paired *t* test (*t* = 7.44, *P* = 0.0005). (*C*) Current–pressure relationships (elicited at −60 mV) from nonsickle (n = 24) and sickle (n = 23) erythrocytes, from seven mice. Symbols are mean ± SEM. (*D*) Mechanocurrents elicited by −130 mmHg from nonsickle and sickle erythrocytes from male and female mice. Two-way ANOVA with Holm–Šidák multiple comparison test (F = 2.23; *P* = 0.14). (*E*) Time required to reach half of the mechanocurrents maximal value (T_0.5_) elicited by −130 mmHg from nonsickle and sickle erythrocytes. Two-tailed Mann–Whitney-test (U = 20, *P* = 2.02^−8^). (*F*) Time constant of deactivation (τ) elicited by the maximum negative pressure from nonsickle and sickle erythrocytes. Two-tailed Mann–Whitney-test (U = 79, *P* = 2.89^−5^). (*G*) % of peak current 30 ms after the −130-mmHg stimulus ends from nonsickle and sickle erythrocytes. Two-tailed Unpaired *t* test (*t* = 2.35, *P* = 0.024). Bars are mean ± SD. Boxplots show the mean, median, and 75^th^ to 25^th^ percentiles. n is denoted above the *x*-axis. Asterisks indicate values significantly different from the control (*P < 0.05 and ***P < 0.001) and n.s. indicates not significantly different. Post hoc *P*-values and source data are available at figshare (40).

Our studies on SCD erythrocytes from humans provided valuable insights into the underlying pathophysiology of this disorder. Next, we shifted our focus to a mouse model of SCD to determine potential strategies for reducing PIEZO1 function in vivo. We measured mechanocurrents in freshly isolated blood samples from young adult Townes mice (i.e., a humanized mouse model of SCD; in which both β-globin alleles carry the sickle mutation, HbSS, hα/hα::β^S^/β^S^). Our results show larger PIEZO1 mechanocurrents in sickle erythrocytes compared to nonsickled cells in a sex- and voltage-independent manner (Fig. 3 *A*–*D* and *SI Appendix*, Fig. S4 *A*–*D*). Likewise, mouse PIEZO1 in sickle erythrocytes takes longer to reach half-maximal activation and deactivation, as well as feature higher residual currents (Fig. 3 *D*–*G* and *SI Appendix*, Fig. S4*E*). Similar to humans, we did not find differences in the latency of responses or current–voltage relationships of PIEZO1 in sickle and nonsickle mouse erythrocytes (*SI Appendix*, Fig. S4 *F* and *G*). Overall, our results demonstrate that the Townes mouse model replicates the enhanced PIEZO1 activity observed in sickle erythrocytes of humans with SCD.

### An EPA-Enriched Diet Restores PIEZO1 Function in Sickle Erythrocytes.

**Fig. 4.**
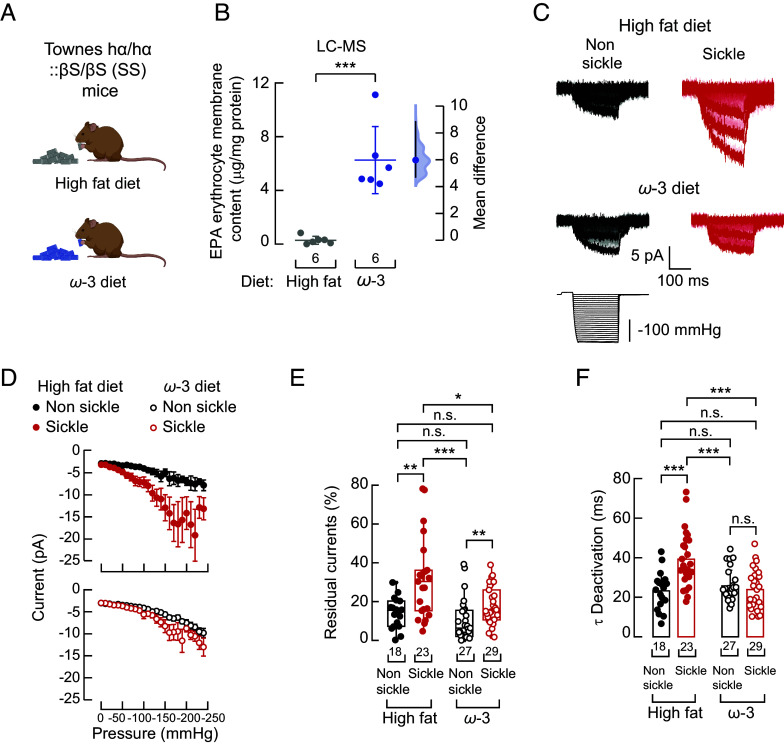
An EPA-enriched diet reduces PIEZO1 currents in sickle erythrocytes. (*A*) Mouse cartoons representing the diets were created with BioRender.com. (*B*) Cumming estimation plot showing the mean difference in the EPA membrane content of erythrocytes from the Townes mouse model of SCD fed with isocaloric high-fat or ω-3 enriched diets, as determined by LC–MS. Two-tailed Unpaired *t* test (*t* = 5.81, *P* = 0.0002). (*C*) Representative inside-out recordings elicited by negative pressure square pulses at a constant voltage of −60 mV from nonsickle and sickle erythrocytes from the Townes mouse model of SCD fed with isocaloric high-fat or ω-3 enriched diets. (*D*, *Top*) Current–pressure relationships (elicited at −60 mV) from nonsickle (n = 18) and sickle (n = 23) erythrocyte from 10 mice fed with a high-fat diet. (*Bottom*) Current–pressure relationships (elicited at −60 mV) from nonsickle (n = 27) and sickle (n = 29) erythrocytes from mice fed with an ω-3 enriched diet. Symbols are mean ± SEM. (*E*) % of peak current 30 ms after the −130-mmHg stimulus ends from nonsickle and sickle erythrocytes of mice fed with isocaloric high-fat or ω-3 enriched diets. Kruskal–Wallis (H = 23.98, *P* = 2.52^−5^) with Dunn’s multiple comparison test. (*F*) Time constant of deactivation (τ) elicited by the maximum negative pressure from nonsickle and sickle erythrocytes of mice fed with isocaloric high-fat or ω-3 enriched diets. Two-way ANOVA with the Tukey multiple comparison test (F = 8.23; *P* = 0.0051). Bars are mean ± SD. Boxplots show the mean, median, and 75^th^ to 25^th^ percentiles. n is denoted above the *x*-axis. Asterisks indicate values significantly different from the control (*P < 0.05, **P < 0.01 and ***P < 0.001) and n.s. indicates not significantly different. Post hoc *P*-values and source data are available at figshare (40).

We have previously shown that dietary fatty acids can modulate the mechanical response of PIEZO channels in vitro, ex vivo, and in vivo (35,36, 37, 44). Specifically, EPA decreases PIEZO1 activity by accelerating channel inactivation (35). Given the elevated PIEZO1 function observed in sickle erythrocytes of the Townes mouse model, we hypothesized that an EPA-enriched diet could restore normal PIEZO1 function. We pair-fed Townes mice for 16 wk with a menhaden oil-enriched diet (high in EPA) or a control isocaloric high-fat diet (high in anhydrous milk fat and soybean oil). EPA accumulates in mouse tissues when its consumption in the diet is increased (36, 45). As expected, Townes mice fed the EPA-enriched diet exhibited higher EPA membrane content than those fed the control diet (Fig. 4 *A* and *B* and Table 1). Remarkably, the EPA-enriched diet reduced PIEZO1 mechanocurrents in sickle erythrocytes to levels similar to those in nonsickle cells (Fig. 4 *C* and *D*). Furthermore, the EPA-enriched diet restored PIEZO1 residual currents, deactivation, and half-maximal activation to levels equivalent to nonsickle erythrocytes (Fig. 4 *E* and *F* and *SI Appendix*, Fig. S5 *A* and *B*). Neither dietary intervention affected the latency of PIEZO1 response to mechanical stimulation (*SI Appendix*, Fig. S5*C*). These results demonstrate that an EPA-enriched diet can effectively restore normal PIEZO1 function in sickle erythrocytes.

**Table 1.** Comparison of erythrocyte membrane fatty acids in SCD mice on high-fat versus ω-3–enriched diets

	High-fat diet	ω-3 diet	
Fatty acids	µg fatty acid/mg erythrocytes Mean ± SD	µg fatty acid/mg erythrocytes Mean ± SD	*P*-value
Dodecanoic acid	0.95 ± 0.53	1.40 ± 1.13	0.3965^*^
Myristic acid	12.54 ± 6.86	16.55 ± 7.86	0.3681^*^
Pentadecanoic acid	3.02 ± 1.45	3.32 ± 1.67	0.7559^*^
Palmitoleic acid	3.70 ± 2.55	102.68 ± 223.61	0.0087^†^
Sapienic acid	3.53 ± 2.39	96.00 ± 209.52	0.0087^†^
Heptadecenoic acid	0.63 ± 0.58	10.74 ± 23.64	0.2616^†^
Heptadecanoic acid	3.07 ± 1.51	3.58 ± 1.85	0.6139^*^
Octadecatetraenoic acid	0.09 ± 0.18	0.14 ± 0.083	0.0647^†^
Linolenic acid (alpha & gamma)	0.16 ± 0.12	0.18 ± 0.097	0.717^*^
Linoleic acid	17.40 ± 11.31	22.26 ± 11.35	0.4749^*^
EPA	0.28 ± 0.30	6.26 ± 2.50	0.0002^*^
Arachidonic acid	189.85 ± 423.52	9.53 ± 4.42	0.3095^†^
Dihomo-γ-linolenic acid	0.13 ± 0.24	0 ± 0	–
Mead acid	0.26 ± 0.21	0.09 ± 0.035	0.1293^*^
Eicosadienoic acid	0.57 ± 0.47	0.44 ± 0.25	0.9361^†^
Eicosenoic acid	0.76 ± 0.55	8.88 ± 19.33	0.2403^†^
Arachidic acid	1.30 ± 0.74	1.50 ± 0.74	0.6577^*^
Docosahexaenoic acid	5.53 ± 4.03	9.73 ± 4.62	0.0649^†^
Docosapentaenoic acid (ω -3)	0.58 ± 0.40	0.76 ± 0.44	0.4732^*^
Docosapentaenoic acid (ω -6)	0.94 ± 0.53	1.29 ± 0.87	0.8182^†^
Docosatetraenoic acid	7.84 ± 17.73	0.12 ± 0.069	0.0082^†^
Docosatrienoic acid	2.29 ± 0.96	2.38 ± 3.93	0.1320^†^
Docosadienoic acid	0.14 ± 0.12	0.18 ± 0.11	0.5577^*^
Docosenoic acid	4.53 ± 3.56	61.14 ± 132.56	0.1320^†^
Docosanoic acid	0.89 ± 0.50	1.49 ± 0.80	0.1538^*^
Nervonic acid	0.14 ± 0.12	0.20 ± 0.077	0.3576^*^
Lignoceric acid	1.16 ± 0.64	1.35 ± 0.88	0.6842^*^
Hexacosenoic acid	0.02 ± 0.012	0.02 ± 0.023	0.9999^†^
Hexacosanoic acid	0.44 ± 0.28	0.36 ± 0.17	0.6495^*^

^*^Two-tailed unpaired *t* test.

^†^Two-tailed Mann–Whitney test.

### EPA-Enriched Diet Reduces Hemolysis, Inflammation, and Hypoxia-Induced Sickling in a Mouse Model of SCD.

**Fig. 5.**
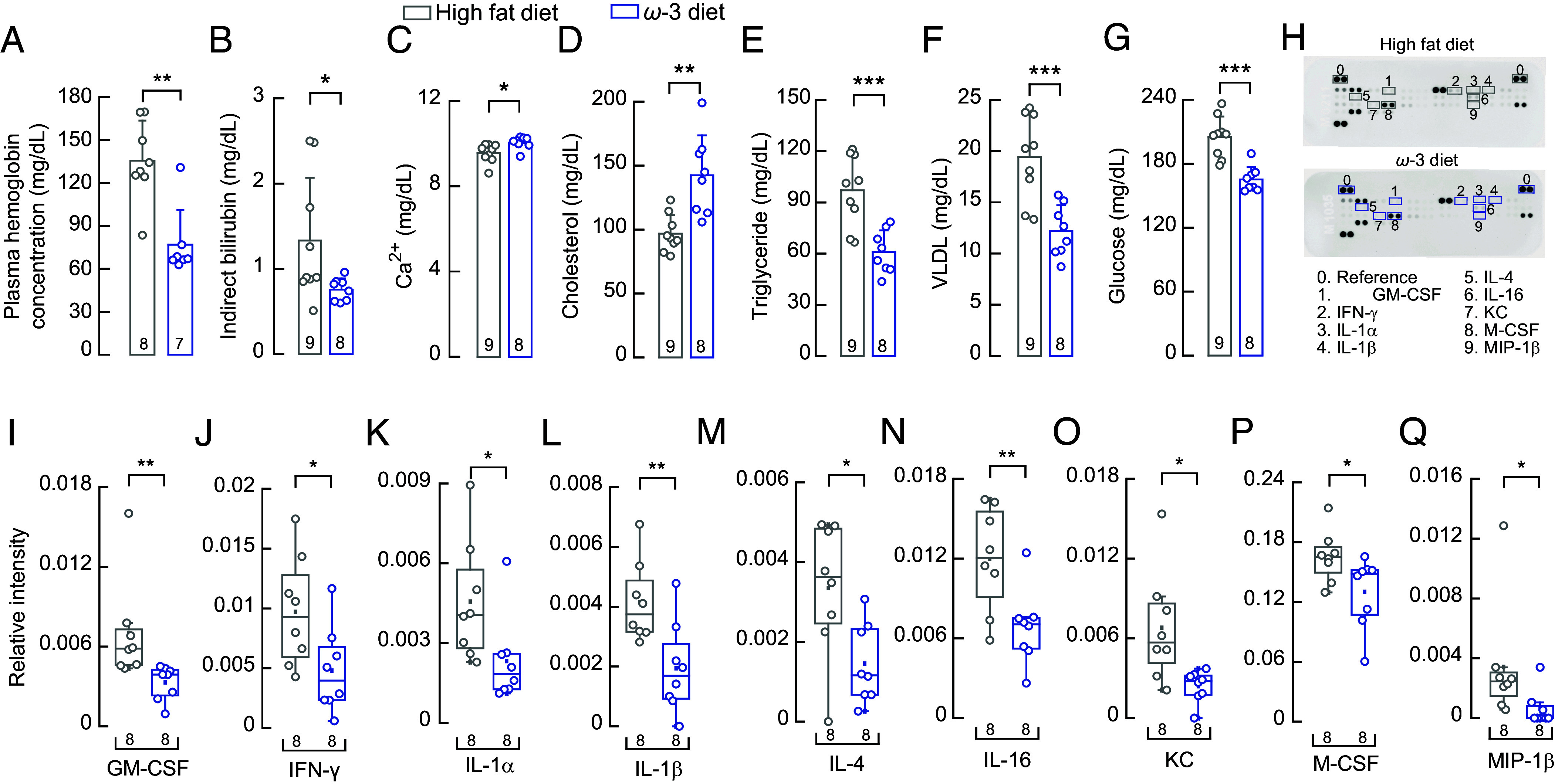
A dietary intervention reduces hemolysis and inflammation in Townes SCD mice. Mice were fed isocaloric high-fat or ω-3–enriched diets (*A*–*Q*) (*A*) Plasma hemoglobin concentration. Two-tailed Mann–Whitney test (U = 52, *P* = 0.0037). (*B*) Serum indirect bilirubin concentration. Two-tailed Mann–Whitney test (U = 12.5, *P* =0.0267). (*C*) Serum calcium concentration. Two-tailed Unpaired *t* test (*t* = 2.622, *P* = 0.0192). (*D*) Serum cholesterol concentration. Two-tailed Unpaired *t* test (*t* = 3.939, *P* = 0.0013). (*E*) Serum triglyceride concentration. Two-tailed Unpaired *t* test (*t* = 4.263, *P* = 0.0007). (*F*) Serum very low-density lipoprotein (VLDL) concentration. Two-tailed Unpaired *t* test (*t* = 4.261, *P* = 0.0007). (*G*) Serum glucose concentration. Two-tailed Unpaired *t* test (*t* = 5.0784, *P* = 0.0001). (*H*) Representative blood serum cytokine profiles and their quantification. (*I*) Relative intensity of serum granulocyte-macrophage colony-stimulating factor (GM-CSF). Two-tailed Mann–Whitney test (U = 1.5, *P* = 0.0016). (*J*) Relative intensity of serum interferon-gamma (IFN-γ). Two-tailed Unpaired *t* test (*t* = 2.390, *P* = 0.0315). (*K*) Relative intensity of serum interleukin-1α (IL-1α). Two-tailed Mann–Whitney test (U = 9, *P* = 0.0148). (*L*) Relative intensity of serum interleukin-1β (IL-1β). Two-tailed Unpaired *t* test (*t* = 3.07, *P* = 0.0083). (*M*) Relative intensity of serum interleukin-4 (IL-4). Two-tailed Unpaired *t* test (*t* = 2.734, *P* = 0.0161). (*N*) Relative intensity of serum interleukin-16 (IL-16). Two-tailed Unpaired *t* test (*t* = 3.011, *P* = 0.0093). (*O*) Relative intensity of serum keratinocyte-derived chemokine CXCL1(KC). Two-tailed Unpaired *t* test (*t* = 2.87, *P* = 0.0123). (*P*) Relative intensity of serum macrophage colony-stimulating factor (M-CSF). Two-tailed Mann–Whitney test (U = 12, *P* = 0.0379). (*Q*) Relative intensity of serum macrophage inflammatory protein-1 beta (MIP-1β). Two-tailed Mann–Whitney test (U = 9, *P* = 0.0165). Bars are mean ± SD. Boxplots show the mean, median, and 75^th^ to 25^th^ percentiles. n is denoted above the *x*-axis. Asterisks indicate values significantly different from the control (*P < 0.05, **P < 0.01, and ***P < 0.001) and n.s. indicates not significantly different. Source data are available at figshare (40).

Based on the relationship between enhanced PIEZO1 function and hemolysis (25), we sought to determine whether decreasing PIEZO1 activity with an EPA-enriched diet could improve hematological parameters associated with SCD pathophysiology. Notably, mice fed the EPA-enriched diet exhibited lower plasma hemoglobin (Fig. 5*A*). Concomitant with the decrease in plasma hemoglobin, we also observed a reduction in indirect bilirubin concentration (as released hemoglobin is converted into bilirubin; Fig. 5*B*). Both results support the notion that restoring PIEZO1’s normal function reduces sickle cell hemolysis. On the other hand, we did not observe differences in parameters such as erythrocyte count, hematocrit, total hemoglobin (i.e., plasma plus intracellular), and mean corpuscular hemoglobin (*SI Appendix*, Fig. S6 *A*–*J*). Our results support that an EPA-enriched diet reduces PIEZO1-mediated hemolysis in SCD.

Since an EPA-enriched diet lowers free hemoglobin (which contributes to oxidative stress and inflammation), we aimed to determine its potential beneficial effects on inflammatory and metabolic markers. To this end, we conducted several analyses, including serum chemistry and cytokine profiling. We found that serum calcium and cholesterol levels rose with the EPA-enriched diet (Fig. 5 *C* and *D*). Importantly, SCD patients often exhibit hypocholesterolemia (46), while hypocalcemia (47) occurs less frequently. Additionally, triglycerides, very low-density lipoprotein, and glucose decreased with the EPA-enriched diet (Fig. 5 *E*–*G*)—an important finding given that these parameters are typically elevated in SCD patients (46). We also report other parameters that showed no significant changes (e.g., potassium, albumin, lactate dehydrogenase; *SI Appendix*, Fig. S6 *K*–*S*). Notably, inflammatory markers contributing to chronic inflammation, endothelial dysfunction, and vaso-occlusive pathology were significantly reduced following the dietary intervention. These included granulocyte-macrophage colony-stimulating factor (GM-CSF), interferon-gamma (IFN-γ), interleukin-1α (IL-1α), interleukin-1β (IL-1β), interleukin-4 (IL-4), interleukin-16 (IL-16), keratinocyte-derived chemokine CXCL1 (KC), macrophage colony-stimulating factor (M-CSF), and macrophage inflammatory protein-1 β (MIP-1β; Fig. 5 *H*–*Q*). Other inflammatory markers decreased after the dietary intervention, albeit not significantly (*SI Appendix*, Fig. S7).

**Fig. 6.**
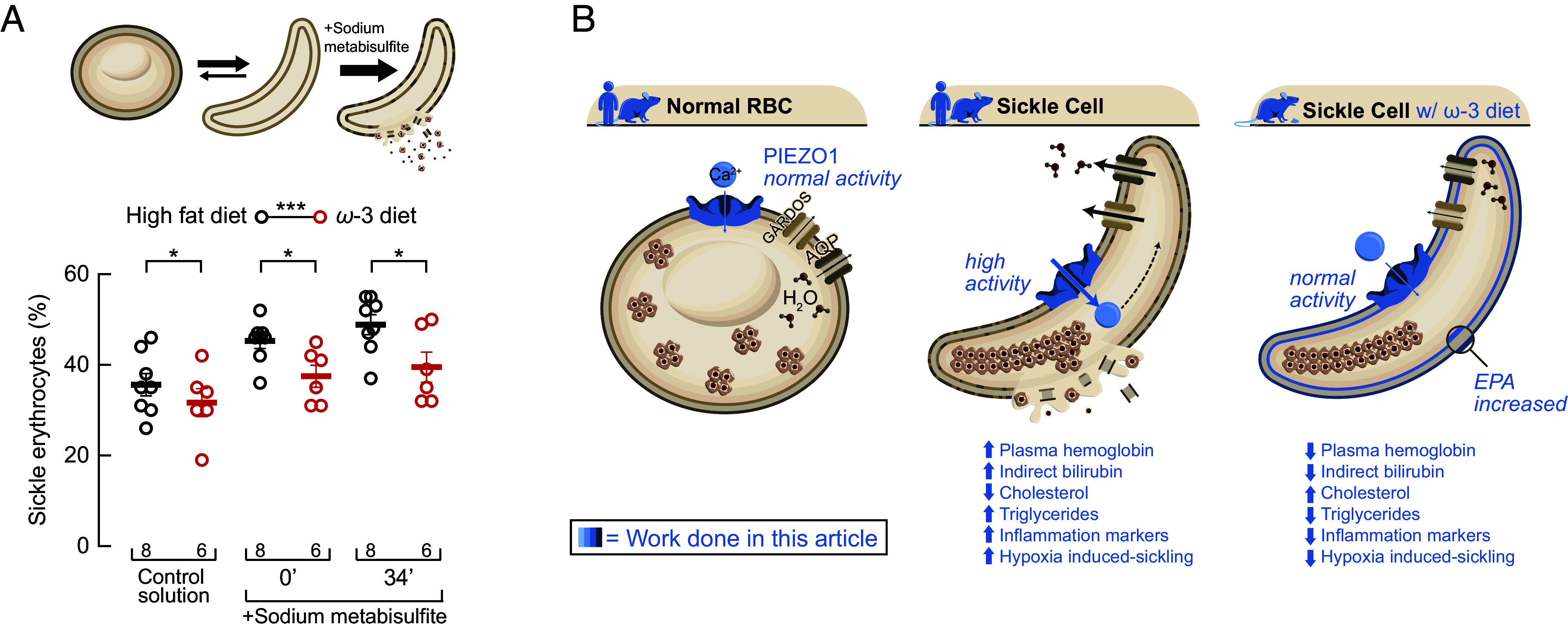
An ω-3-enriched diet reduces erythrocyte hypoxia-induced sickling. (*A*, *Top*) Representative cartoon of erythrocyte sickling following sodium metabisulfite–induced hypoxic stress. (*Bottom*) Quantification of sickle erythrocytes from sickle cell mice fed a high-fat or ω-3-enriched diets. Percent sickle erythrocytes under control conditions and after 1% sodium metabisulfite treatment (0 and 34 min). Two-way ANOVA with Holm–Šidák multiple comparison test (F = 6.723; *P* = 0.0002). Lines are mean ± SD. Asterisks indicate values significantly different from the control (*P < 0.05 and ***P < 0.001). Post hoc *P*-values and source data are available at figshare (40). (*B*) Proposed mechanism: In normal red blood cells (RBCs), PIEZO1 activity supports balanced Ca^2+^ influx and hydration. In sickle RBCs, PIEZO1 is hyperactive, directly enhancing Ca^2+^ entry, Gárdos channel–mediated dehydration, sickling, and hemolysis; downstream, this manifests as higher bilirubin, triglycerides, and inflammatory markers. An ω-3–enriched diet increases EPA in the RBC membrane, restores near-normal PIEZO1 activity, reducing hypoxia-induced sickling and improving biochemical markers (reduced plasma hemoglobin, bilirubin, triglycerides, and inflammation; increased cholesterol).

To functionally validate the effects of the dietary intervention, we performed erythrocyte morphology analysis and sickling assays under deoxygenated conditions. To this end, erythrocytes from peripheral blood were challenged with sodium metabisulfite, an oxygen scavenger commonly used in clinical laboratories. Consistent with our hypothesis, erythrocytes from EPA-fed SCD mice exhibited a significant reduction in hypoxia-induced sickling compared with those from mice fed a control high-fat diet (Fig. 6*A*). Together, these findings demonstrate that an EPA-enriched diet confers multiple benefits by reducing hemolysis, stabilizing serum lipid profiles, lowering inflammatory markers, and improving erythrocyte deformability under hypoxic stress.

## Discussion

Our study provides insights into the role of PIEZO1 in SCD, highlighting the therapeutic potential of targeting this mechanosensitive ion channel to alleviate some of the symptoms associated with this disease. We demonstrated that PIEZO1 currents are enhanced in both humans and a humanized mouse model with SCD in a sex-independent manner. This functional upregulation mirrors the GOF mutation in PIEZO1 that causes hemolytic anemia, suggesting a common pathological mechanism involving increased cation permeability and erythrocyte dehydration.

Multiple pieces of indirect evidence suggest that PIEZO1 might play a role in SCD. For instance, 1) Cahalan et al. showed that PIEZO1 transduces mechanical forces to regulate mouse erythrocyte volume and calcium concentration, and erythrocytes lacking PIEZO1 fail to permeate calcium in response to mechanical stimuli (24); 2) Vaisey et al. recorded stretch-dependent single-channel currents in a mouse erythrocyte with a conductance matching that of PIEZO1 in transfected cells (48); 3) GOF mutations in PIEZO1 cause hereditary hemolytic anemia in both humans and mouse models (22, 25–28); and 4) Chemical sensitization of PIEZO1 with Yoda1 increases the sickling propensity of human erythrocytes (49). However, no direct evidence showed whether PIEZO1 function is altered in SCD. Our electrophysiological characterization of mechanically activated currents in erythrocytes from humans and mice with SCD directly demonstrates that the function of PIEZO1 is increased in this context. This enhancement likely contributes to the nonspecific cationic conductance known as Psickle, which is activated downstream of hemoglobin polymerization (1). Our findings, summarized in Fig. 6*B*, demonstrate that inhibiting PIEZO1 function could decrease the abnormal calcium influx that enhances the Gárdos channel activity, leading to erythrocyte dehydration. Therefore, by targeting PIEZO1, we could potentially disrupt this pathological pathway and improve the clinical outcomes for SCD patients.

An important question to consider is which factors in erythrocyte sickling could contribute to increased PIEZO1 function in vivo. Erythrocytes experience significant macroscopic shape changes upon sickling, transitioning from their normal biconcave disc shape to elongated and rigid forms (1). Since PIEZO1 activity and conformation can be modulated by global curvature (50,51, 52), this dramatic change in morphology could expose PIEZO1 to membrane curvatures that favor its opening. The erythrocyte’s membrane also undergoes significant mesoscopic alterations. Membranes of erythrocytes from patients with SCD are characterized by unbalanced phospholipid remodeling pathways (i.e., Lands’ cycle) (53), abnormal membrane fatty acid composition (31), loss of phospholipid asymmetry (i.e., exposure of phosphatidylserine) (54), and activation of sphingomyelinase and enrichment of its downstream product ceramide (55). Many of these factors are known to increase PIEZO1 channel function in other cell types. For instance, *ω*-6 polyunsaturated fatty acid enrichment enhances PIEZO1 function in human microvascular endothelial cells (35), surface flip-flop of phosphatidylserine activates PIEZO1 in human myotubes (56), and increased sphingomyelinase activity and ceramide content enhance the prolonged activation of PIEZO1 in mouse mesenteric endothelial cells (57). Our study lays the foundation for further examination of these pathways to define the mechanisms that enhance PIEZO1 function in SCD, potentially leading to novel treatments.

One of our key findings is that EPA significantly reduces the function of PIEZO1 in sickle erythrocytes. This reduction leads to decreased hemolysis (as evidenced by lower plasma hemoglobin and indirect bilirubin), reduced hypoxia-induced sickling, and lower inflammatory markers. The significant decrease of inflammatory markers, including GM-CSF, IFN-γ, IL-1α, IL-1β, IL-4, IL-16, KC, M-CSF, and MIP-1β, underscores the anti-inflammatory effect of EPA. Chronic inflammation is a hallmark of SCD, contributing to endothelial dysfunction and vaso-occlusive crises (1, 4). By lowering these inflammatory markers, EPA may help alleviate some of the debilitating symptoms of SCD. It is still unclear whether these broader physiological benefits are due solely to the inhibition of PIEZO1 function or if they also result from EPA pleiotropic effects on the vascular system and its anti-inflammatory properties (45, 58, 59).

A clinical trial of the Gárdos channel inhibitor Senicapoc in SCD patients reduced hemolysis but was terminated early due to insufficient clinical benefits (60, 61). Other clinical trials showed that an *ω*-3 dietary intervention, consisting of more docosahexaenoic acid (DHA) than EPA, reduced the frequency of painful vaso-occlusive crises (62,64, 65). However, the clinical and biochemical evidence remains inconclusive to date (64). Our study in mice identified EPA (but not DHA, Table 1) as a key player in downregulating PIEZO1 function. This distinction is important because we and others have demonstrated that DHA has the opposite effect on PIEZO1 function by slowing inactivation (i.e., increasing function), compared to EPA (35, 66). We propose that increasing the EPA content in erythrocytes, alongside existing treatments for SCD, could improve the effectiveness of clinical interventions. Future research should focus on clinical trials for evaluating the efficacy of a highly purified form of EPA, which was recently approved by the FDA (i.e., icosapent ethyl or Vascepa) (67,68, 69), in SCD patients, as well as investigate other potential inhibitors of PIEZO1 function.

## Materials and Methods

### Ethics Approval.

The participation of human donors was approved by the Institutional Review Board of the University of Tennessee Health Science Center (UTHSC; IRB 20-07604-XP). All participants provided written informed consent. Mice procedures described below were reviewed and approved by the UTHSC Institutional Animal Care and Use Committee (UTHSC IACUC number: Protocol #19-0084 and #22-0320) and the University of Texas Health Science Center (UT Houston; AWC-23-0093). All methods were carried out following approved guidelines.

### Human Samples.

Patients between ages 18 to 65, with confirmed diagnoses of sickle cell anemia (11 HbSS and one HbSβ^0^ thalassemia), who had no severe pain episodes requiring medical contact during the preceding 4 wk donated a blood sample while in noncrisis, steady states. Subjects were recruited during routine clinic visits at the Methodist Comprehensive Sickle Cell Center in Memphis, Tennessee. Participants were excluded if they were pregnant, on anticoagulant therapy, or if less than 3 mo had passed since their last transfusion. Blood was collected in sterile BD vacutainer blood collection tubes with K2 ethylenediaminetetraacetic acid (EDTA) and used for experiments within 4 h of collection.

### Mice.

Male and female Townes transgenic HbSS (hα/hα::β^S^/β^S^; sickling) mice were obtained from the Jackson laboratory or bred in-house from heterozygous breeders (hα/hα::β^A^/β^S^; coming from strain #013071) (70). C57BL/6J mice (WT) were obtained from The Jackson Laboratory (Stock No. 000664). *Piezo1* GOF (R2482H; in the hematopoietic system) mice were generated by breeding *Piezo1*^cx/cx^ (25) with *Vav1-cre* (The Jackson Laboratory, stock# 018968). GOF *Piezo1* (R2482H) mice were generated and maintained on the C57BL/6 background (these animals were backcrossed at least 10 generations to C57BL/6). Littermates were used as control animals for experiments involving GOF *Piezo1* mice. Adult (2- to 6-mo-old) mice were housed with a 12 h light/dark cycle with food and water ad libitum.

### Blood Collection.

*For electrophysiology*. Blood collected through the tail vein puncture was diluted in the solution used for electrophysiological experiments and used within 4 h of collection. Blood collected through cardiac puncture was collected in K2 EDTA tubes and used within 4 h of collection. For GOF *Piezo1* or their WT littermates drawn blood was collected, in UT Southwestern Medical Center in Dallas, into tubes containing acid-citrate-dextrose buffer (Sigma-Aldrich C3821), centrifuged at 100 g for 15 min at room temperature. The supernatant, and buffy coat were discarded, and the erythrocyte pellet was resuspended and washed with PBS two-three times. After the final wash, erythrocytes were diluted in 0.9% NaCl saline, kept on ice packs, and shipped overnight to UT Houston. These erythrocytes were used for electrophysiological recordings within 18 h of collection. *For hematological parameters*. Blood was collected in a microsample tube EDTA K3E (Sarstedt Inc., Cat#41.1504.005) by cardiac puncture. For plasma collection, the blood was centrifuged at 2,000 g for 15 min at 4 °C. The plasma was separated and stored at −80 °C. The buffy coat was discarded. The bottom-packed erythrocytes were collected and stored at −80 °C. For serum collection, mouse blood was collected in a microsample tube serum CAT (Sarstedt Inc., Cat #41.1392.105). The serum microsample tube was left in an upright position undisturbed for 20 min at room temperature allowing the blood to clot. Then, the blood was centrifuged at 1,500 g for 15 min at 4 °C. Serum was separated, transferred into new microcentrifuge tubes, stored at −80 °C, and thawed at the time of assay.

### Electrophysiology.

Patch-clamp recordings were performed in the inside-out configuration in the voltage-clamp mode. The bath solution contained 140 mM KCl, 6 mM NaCl, 2 mM CaCl_2_, 1 mM MgCl_2_, 10 mM glucose, and 10 mM HEPES (pH 7.4), while the pipette solution contained 140 mM NaCl, 6 mM KCl, 2 mM CaCl_2_, 1 mM MgCl_2_, 10 mM glucose, and 10 mM HEPES (pH 7.4). Pipettes were made of borosilicate glass (Sutter Instruments) and fire-polished to a resistance between 8 and 10 MΩ before use. Recordings were performed using the voltage-clamp (constant −60 mV, unless otherwise noted), sampled at 100 kHz, and low pass filtered at 2 kHz using a MultiClamp 700 B amplifier and Clampex (Molecular Devices, LLC). Yoda1 (Tocris Bioscience) and GsMTx-4 (Abcam) stocks were prepared using DMSO and water, respectively, and diluted in the pipette solution to face the membrane from the outer leaflet. Leak currents before mechanical stimulation were subtracted offline from the current traces with ClampFit (Molecular Devices, LLC). Recordings with leak currents >50 pA and patches with giga-seals that did not withstand at least five consecutive steps of mechanical stimulation were excluded from analyses.

### Mechanical Stimulation.

*For pressure-clamp assays*, Excised membrane patches were mechanically stimulated with negative pressure applied through the patch pipette using a High-Speed Pressure Clamp (ALA Scientific), which was automated using a MultiClamp 700B amplifier through Clampex. Inside-out patches were probed using a square-pulse protocol consisting of a 50 ms 10-mmHg prepulse, immediately followed by incremental pressure steps (−10 mmHg), each lasting 200 ms in 1-s intervals. *For current–voltage (IV) relationships*, a square-pulse protocol consisting of a 1-s −120 mmHg pressure step was applied every 10 s at a voltage ranging from −60 to 60 mV.

### Diet Supplementation.

HbSS (6 to 8 wk old) mice were pair-fed for 16 wk with a high-fat (anhydrous milk fat supplemented, Dyets # 105181) modified AIN-93G purified rodent diet with 59% fat-derived calories from anhydrous milk fat (kcal/kg): casein (716), L-cystine (12), maltose dextrin (502), cornstarch (818.76), anhydrous milk fat (2,430), soybean oil (630), mineral mix (# 210025; 30.8), and vitamin mix (#310025; 38.7) or an ω-3 (enriched in EPA; Dyets Inc. #105183) modified AIN 93G enriched diet with 59% fat derived calories from menhaden oil (kcal/kg): casein (716), L-cystine (12), maltose dextrin (502), cornstarch (818.76), menhaden oil (2,430), soybean oil (630), mineral mix (#210025; 30.8), and vitamin mix (#310025; 38.7).

### Liquid Chromatography–Mass Spectrometry.

The bottom-packed erythrocytes collected from the blood of mice fed with high fat or ω-3 diet were shipped to Wayne State University. Membrane (total minus free) fatty acids were quantified at the Lipidomics Core Facility at Wayne State University.

### Plasma Hemoglobin.

Mice plasma hemoglobin concentration was measured with the Quantichrom™ Hemoglobin Assay Kit (BioAssay systems, Cat #DIHB-250) following the manufacturer’s instructions.

### Serum Chemistry Analyses.

Mice serum chemistry parameters were obtained using a Beckman Coulter AU480 Automated analyzer at the Center of Comparative Medicine at Baylor College of Medicine.

### Mouse Cytokine Profile Assay.

Serum cytokine profiles were determined with the Mouse Cytokine Array Panel kit (R&D Systems, Cat #ARY006). 100 μL of serum sample was used for each array. The serum sample was diluted in array buffers and mixed with reconstituted mouse cytokine array Panel A detection antibody cocktail. The sample antibody mixture was incubated for 1 h at room temperature and was added to the nitrocellulose membrane. The membrane was incubated overnight at 4 °C. Streptavidin-HRP (1:2,000) was added after washes to remove unbound materials, and a chemiluminescent reagents mix was used for detecting cytokine binding. Membranes were imaged in a ChemiDoc Touch Imaging System (v6.1.0 build 7; Bio-Rad) for chemiluminescence.

### Plasma Haptoglobin.

Plasma samples were thawed at the time of assay and processed according to the haptoglobin enzyme-linked immunosorbent assay (ELISA) kit (Aviva Systems biology, Cat #OKIA00095). The plasma samples were diluted and treated in microtiter wells coated with antihaptoglobin (anti-HPT) antibodies. Anti-HPT antibodies conjugated with horseradish peroxidase were added after washing. Then, a chromogenic substrate solution was added. Sample absorbance was measured at 450 nm with an Infinite M200 PRO NanoQuant microplate reader (Tecan).

### Hematological Indices.

Whole blood samples were processed within 30 min of collection on a VETSCAN® HM5 Hematology Analyzer (Zoetis), per the manufacturer’s instructions to obtain the following parameters: erythrocyte count, hematocrit, total hemoglobin (i.e., plasma plus intracellular), mean corpuscular hemoglobin, and mean corpuscular volume.

### Sodium Metabisulfite-Induced Sickling Assay.

Mouse blood was collected from the tail vein and immediately diluted 1:500 in a control solution (pH 7.4; 140 mM NaCl, 6 mM KCl, 2 mM CaCl_2_, 1 mM MgCl_2_, 10 mM glucose, 10 mM 2-(2-hydroxyethyl)piperazin-1-yl)ethanesulfonic acid (HEPES). A sodium metabisulfite working solution (from a freshly prepared stock) was prepared in the same buffer and added to the diluted blood to achieve a final concentration of 1% (w/v) sodium metabisulfite. The suspension was then further diluted 1:3 with Hayem’s diluting fluid (Ricca Chemical, Cat. 3500-16), loaded into a hemocytometer (Fisher Scientific, Cat. 0267151B), and erythrocytes were counted at 0 and 34 min after metabisulfite addition. The percentage of sickled erythrocytes was calculated as 100 × (number of morphologically sickled cells ÷ total erythrocytes counted).

### Data Analysis, Statistics, and Reproducibility.

Data were plotted using OriginPro (2018 v:b9.51.195; OriginLab Corp.). The time constant of deactivation τ was obtained by fitting a single exponential function (**1**):

[1] $$f_{\left(t\right)}=\sum_{i=1}^{n}A_{i}*e^{\frac{-t}{\tau_{i}}}+C,$$

where A = amplitude, τ = time constant, and the constant y-offset C for each component i. All boxplots show mean (square), median (bisecting line), bounds of the box (75th to 25th percentiles), and outlier range with 1.5 coefficient (whiskers). Statistical analyses were performed using GraphPad InStat software (version 3.10; GraphPad Software Inc.). We used the Kolmogorov and Smirnov method to determine data distribution, as well as Bartlett’s test to determine differences between SD. Individual tests are described in each of the figure legends. No statistical method was used to predetermine the sample size. No data were excluded from the analyses. The experiments were not randomized. The investigators were blind to genotype and treatment whenever possible. Experiments were performed at least three times on different days from different/independent preparations.

## Supplementary Material

Appendix 01 (PDF)

## Data Availability

The source data underlying figures and supplementary figures are provided as a Source Data file and deposited at figshare (40).

## References

[1] G. J. Kato , Sickle cell disease. Nat. Rev. Dis. Primers **4**, 18010 (2018). 29542687 10.1038/nrdp.2018.10

[2] C. f. D. C. a. Prevention, Data and statistics on sickle cell disease. Retrieved 12 August 2024.

[3] L. National Heart, and Blood Institute, Sickle cell disease: Treatment. Retrieved 12 August 2024.

[4] M. T. Gladwin, G. J. Kato, E. M. Novelli, Sickle Cell Disease (McGraw-Hill Education, New York, NY, 2021).

[5] L. Pauling , Sickle cell anemia a molecular disease. Science **110**, 543–548 (1949). 15395398 10.1126/science.110.2865.543

[6] W. A. Eaton, Linus Pauling and sickle cell disease. Biophys. Chem. **100**, 109–116 (2003). 12646356 10.1016/s0301-4622(02)00269-7

[7] T. Jang , Vaso-occlusive crisis in sickle cell disease: A vicious cycle of secondary events. J. Transl. Med. **19**, 397 (2021). 34544432 10.1186/s12967-021-03074-z PMC8454100

[8] D. Manwani, P. S. Frenette, Vaso-occlusion in sickle cell disease: Pathophysiology and novel targeted therapies. Blood **122**, 3892–3898 (2013). 24052549 10.1182/blood-2013-05-498311 PMC3854110

[9] R. B. Francis Jr., L. J. Haywood, Elevated immunoreactive tumor necrosis factor and interleukin-1 in sickle cell disease. J. Natl. Med. Assoc. **84**, 611–615 (1992). 1629925 PMC2571695

[10] B. Keikhaei , Altered levels of pro-inflammatory cytokines in sickle cell disease patients during vaso-occlusive crises and the steady state condition. Eur. Cytokine Netw. **24**, 45–52 (2013). 23608554 10.1684/ecn.2013.0328

[11] R. P. Santiago , Serum haptoglobin and hemopexin levels are depleted in pediatric sickle cell disease patients. Blood Cells Mol. Dis. **72**, 34–36 (2018). 30033157 10.1016/j.bcmd.2018.07.002 PMC6389271

[12] C. T. Noguchi, A. N. Schechter, The intracellular polymerization of sickle hemoglobin and its relevance to sickle cell disease. Blood **58**, 1057–1068 (1981). 7030432

[13] A. Hannemann, D. C. Rees, S. Tewari, J. S. Gibson, Cation homeostasis in red cells from patients with sickle cell disease heterologous for HbS and HbC (HbSC genotype). EBioMedicine **2**, 1669–1676 (2015). 26870793 10.1016/j.ebiom.2015.09.026 PMC4740305

[14] F. A. Kuypers, Hemoglobin s polymerization and red cell membrane changes. Hematol. Oncol. Clin. North Am. **28**, 155–179 (2014). 24589260 10.1016/j.hoc.2013.12.002

[15] J. W. Eaton, T. D. Skelton, H. S. Swofford, C. E. Kolpin, H. S. Jacob, Elevated erythrocyte calcium in sickle cell disease. Nature **246**, 105–106 (1973). 4585849 10.1038/246105a0

[16] C. Brugnara, L. de Franceschi, S. L. Alper, Inhibition of Ca(2+)-dependent K+ transport and cell dehydration in sickle erythrocytes by clotrimazole and other imidazole derivatives. J. Clin. Invest. **92**, 520–526 (1993). 8326017 10.1172/JCI116597 PMC293641

[17] H. Izumo, S. Lear, M. Williams, R. Rosa, F. H. Epstein, Sodium-potassium pump, ion fluxes, and cellular dehydration in sickle cell anemia. J. Clin. Invest. **79**, 1621–1628 (1987). 3034977 10.1172/JCI112998 PMC424484

[18] R. M. Bookchin, V. L. Lew, Effect of a “sickling pulse” on calcium and potassium transport in sickle cell trait red cells. J. Physiol. **312**, 265–280 (1981). 7264994 10.1113/jphysiol.1981.sp013628 PMC1275553

[19] V. L. Lew, R. M. Bookchin, Ion transport pathology in the mechanism of sickle cell dehydration. Physiol. Rev. **85**, 179–200 (2005). 15618480 10.1152/physrev.00052.2003

[20] V. L. Lew, O. E. Ortiz, R. M. Bookchin, Stochastic nature and red cell population distribution of the sickling-induced Ca2+ permeability. J. Clin. Invest. **99**, 2727–2735 (1997). 9169503 10.1172/JCI119462 PMC508119

[21] C. Milligan , A non-electrolyte haemolysis assay for diagnosis and prognosis of sickle cell disease. J. Physiol. **591**, 1463–1474 (2013). 23297308 10.1113/jphysiol.2012.246579 PMC3607166

[22] J. Albuisson , Dehydrated hereditary stomatocytosis linked to gain-of-function mutations in mechanically activated PIEZO1 ion channels. Nat. Commun. **4**, 1884 (2013). 23695678 10.1038/ncomms2899 PMC3674779

[23] R. Zarychanski , Mutations in the mechanotransduction protein PIEZO1 are associated with hereditary xerocytosis. Blood **120**, 1908–1915 (2012). 22529292 10.1182/blood-2012-04-422253 PMC3448561

[24] S. M. Cahalan , Piezo1 links mechanical forces to red blood cell volume. Elife **4**, 1–12 (2015). 10.7554/eLife.07370 PMC4456639 26001274

[25] S. Ma , Common PIEZO1 allele in African populations causes RBC dehydration and attenuates *Plasmodium* infection. Cell **173**, 443–455.e412 (2018). 29576450 10.1016/j.cell.2018.02.047 PMC5889333

[26] I. Andolfo , Multiple clinical forms of dehydrated hereditary stomatocytosis arise from mutations in PIEZO1. Blood **121**, 3925–3935, s1–s12 (2013). 23479567 10.1182/blood-2013-02-482489

[27] N. M. Archer , Hereditary xerocytosis revisited. Am. J. Hematol. **89**, 1142–1146 (2014). 25044010 10.1002/ajh.23799 PMC4237618

[28] B. E. Shmukler , Dehydrated stomatocytic anemia due to the heterozygous mutation R2456H in the mechanosensitive cation channel PIEZO1: A case report. Blood Cells Mol. Dis. **52**, 53–54 (2014). 23973043 10.1016/j.bcmd.2013.07.015

[29] B. F. Cameron, P. Smariga, Calcium exchange and calcium-related effects in normal and sickle cell anemia erythrocytes. Prog. Clin. Biol. Res. **20**, 105–122 (1978). 148652

[30] B. N. Erickson, H. H. Williams, F. C. Hummel, P. Lee, I. G. Macy, The lipid and mineral distribution of the serum and erythrocytes in the hemolytic and hypochromic anemias of childhood. J. Biol. Chem. **118**, 569–598 (1937).

[31] W. E. Connor , Abnormal phospholipid molecular species of erythrocytes in sickle cell anemia. J. Lipid Res. **38**, 2516–2528 (1997). 9458275

[32] H. Ren , Blood mononuclear cells and platelets have abnormal fatty acid composition in homozygous sickle cell disease. Ann. Hematol. **84**, 578–583 (2005). 15809883 10.1007/s00277-005-1023-7

[33] S. Zorca , Lipid levels in sickle-cell disease associated with haemolytic severity, vascular dysfunction and pulmonary hypertension. Br. J. Haematol. **149**, 436–445 (2010). 20230401 10.1111/j.1365-2141.2010.08109.x PMC3212812

[34] D. J. VanderJagt , Phase angle correlates with n-3 fatty acids and cholesterol in red cells of Nigerian children with sickle cell disease. Lipids Health Dis. **2**, 2 (2003). 12773201 10.1186/1476-511X-2-2 PMC156645

[35] L. O. Romero , Dietary fatty acids fine-tune Piezo1 mechanical response. Nat. Commun. **10**, 1200 (2019). 30867417 10.1038/s41467-019-09055-7 PMC6416271

[36] S. Ma , Excessive mechanotransduction in sensory neurons causes joint contractures. Science **379**, 201–206 (2023). 36634173 10.1126/science.add3598 PMC10163824

[37] L. O. Romero , Linoleic acid improves PIEZO2 dysfunction in a mouse model of Angelman syndrome. Nat. Commun. **14**, 1167 (2023). 36859399 10.1038/s41467-023-36818-0 PMC9977963

[38] S. Chien, S. Usami, J. F. Bertles, Abnormal rheology of oxygenated blood in sickle cell anemia. J. Clin. Invest. **49**, 623–634 (1970). 5443167 10.1172/JCI106273 PMC322516

[39] G. R. Serjeant, B. E. Serjeant, P. F. Milner, The irreversibly sickled cell; A determinant of haemolysis in sickle cell anaemia. Br. J. Haematol. **17**, 527–533 (1969). 5362290 10.1111/j.1365-2141.1969.tb01403.x

[40] L. O. Romero , Enhanced PIEZO1 function contributes to the pathogenesis of sickle cell disease. Figshare. 10.6084/m9.figshare.29321084.v1. Deposited 18 September 2025. PMC12519228 41037639

[41] R. Syeda , Chemical activation of the mechanotransduction channel Piezo1. Elife **4**, 1–11 (2015). 10.7554/eLife.07369 PMC4456433 26001275

[42] C. Bae, F. Sachs, P. A. Gottlieb, The mechanosensitive ion channel Piezo1 is inhibited by the peptide GsMTx4. Biochemistry **50**, 6295–6300 (2011). 21696149 10.1021/bi200770q PMC3169095

[43] C. Bae, R. Gnanasambandam, C. Nicolai, F. Sachs, P. A. Gottlieb, Xerocytosis is caused by mutations that alter the kinetics of the mechanosensitive channel PIEZO1. Proc. Natl. Acad. Sci. U.S.A. **110**, E1162–E1168 (2013). 23487776 10.1073/pnas.1219777110 PMC3606986

[44] L. O. Romero , A dietary fatty acid counteracts neuronal mechanical sensitization. Nat. Commun. **11**, 2997 (2020). 32561714 10.1038/s41467-020-16816-2 PMC7305179

[45] R. Caires , Genetic- and diet-induced ω-3 fatty acid enrichment enhances TRPV4-mediated vasodilation in mice. Cell Rep. **40**, 111306 (2022). 36070688 10.1016/j.celrep.2022.111306 PMC9498980

[46] A. Yalcinkaya, S. Unal, Y. Oztas, Altered HDL particle in sickle cell disease: Decreased cholesterol content is associated with hemolysis, whereas decreased apolipoprotein A1 is linked to inflammation. Lipids Health Dis. **18**, 225 (2019). 31861992 10.1186/s12944-019-1174-5 PMC6924024

[47] C. Antwi-Boasiako , Total serum magnesium levels and calcium-to-magnesium ratio in sickle cell disease. Medicina (Kaunas) **55**, 1–8 (2019). 10.3390/medicina55090547 PMC6780276 31470666

[48] G. Vaisey, P. Banerjee, A. J. North, C. A. Haselwandter, R. MacKinnon, Piezo1 as a force-through-membrane sensor in red blood cells. Elife **11**, 1–21 (2022). 10.7554/eLife.82621 PMC9750178 36515266

[49] E. Nader , Piezo1 activation augments sickling propensity and the adhesive properties of sickle red blood cells in a calcium-dependent manner. Br. J. Haematol. **202**, 657–668 (2023). 37011913 10.1111/bjh.18799

[50] A. H. Lewis, J. Grandl, Mechanical sensitivity of Piezo1 ion channels can be tuned by cellular membrane tension. Elife **4**, 1–17 (2015). 10.7554/eLife.12088 PMC4718726 26646186

[51] Y. C. Lin , Force-induced conformational changes in PIEZO1. Nature **573**, 230–234 (2019). 31435018 10.1038/s41586-019-1499-2 PMC7258172

[52] S. Yang , Membrane curvature governs the distribution of Piezo1 in live cells. Nat. Commun. **13**, 7467 (2022). 36463216 10.1038/s41467-022-35034-6 PMC9719557

[53] H. Wu , Hypoxia-mediated impaired erythrocyte Lands’ cycle is pathogenic for sickle cell disease. Sci. Rep. **6**, 29637 (2016). 27436223 10.1038/srep29637 PMC4951653

[54] F. A. Kuypers, K. de Jong, The role of phosphatidylserine in recognition and removal of erythrocytes. Cell Mol. Biol. (Noisy-le-grand) **50**, 147–158 (2004). 15095785

[55] A. O. Awojoodu , Acid sphingomyelinase is activated in sickle cell erythrocytes and contributes to inflammatory microparticle generation in SCD. Blood **124**, 1941–1950 (2014). 25075126 10.1182/blood-2014-01-543652 PMC4168349

[56] M. Tsuchiya , Cell surface flip-flop of phosphatidylserine is critical for PIEZO1-mediated myotube formation. Nat. Commun. **9**, 2049 (2018). 29799007 10.1038/s41467-018-04436-w PMC5967302

[57] J. Shi , Sphingomyelinase disables inactivation in endogenous PIEZO1 channels. Cell Rep. **33**, 108225 (2020). 33027663 10.1016/j.celrep.2020.108225 PMC7539531

[58] J. Endo, M. Arita, Cardioprotective mechanism of omega-3 polyunsaturated fatty acids. J. Cardiol. **67**, 22–27 (2016). 26359712 10.1016/j.jjcc.2015.08.002

[59] E. F. Wiest, M. T. Walsh-Wilcox, M. Rothe, W. H. Schunck, M. K. Walker, Dietary omega-3 polyunsaturated fatty acids prevent vascular dysfunction and attenuate cytochrome P4501A1 expression by 2, 3, 7, 8-tetrachlorodibenzo-P-dioxin. Toxicol. Sci. **154**, 43–54 (2016). 27492226 10.1093/toxsci/kfw145 PMC5091366

[60] K. I. Ataga , Improvements in haemolysis and indicators of erythrocyte survival do not correlate with acute vaso-occlusive crises in patients with sickle cell disease: A phase III randomized, placebo-controlled, double-blind study of the Gardos channel blocker senicapoc (ICA-17043). Br. J. Haematol. **153**, 92–104 (2011). 21323872 10.1111/j.1365-2141.2010.08520.x

[61] K. I. Ataga , Efficacy and safety of the Gardos channel blocker, senicapoc (ICA-17043), in patients with sickle cell anemia. Blood **111**, 3991–3997 (2008). 18192510 10.1182/blood-2007-08-110098

[62] A. A. Daak , Double-blind, randomized, multicenter phase 2 study of SC411 in children with sickle cell disease (SCOT trial). Blood Adv. **2**, 1969–1979 (2018). 30097463 10.1182/bloodadvances.2018021444 PMC6093734

[63] A. A. Daak , Effect of omega-3 (n-3) fatty acid supplementation in patients with sickle cell anemia: Randomized, double-blind, placebo-controlled trial. Am. J. Clin. Nutr. **97**, 37–44 (2013). 23193009 10.3945/ajcn.112.036319

[64] A. A. Daak, M. A. Lopez-Toledano, M. M. Heeney, Biochemical and therapeutic effects of omega-3 fatty acids in sickle cell disease. Complement. Ther. Med. **52**, 102482 (2020). 32951732 10.1016/j.ctim.2020.102482

[65] A. Tomer , Reduction of pain episodes and prothrombotic activity in sickle cell disease by dietary n-3 fatty acids. Thromb. Haemost. **85**, 966–974 (2001). 11434703

[66] P. Ridone , Disruption of membrane cholesterol organization impairs the activity of PIEZO1 channel clusters. J. Gen. Physiol. **152**, 1–29 (2020). 10.1085/jgp.201912515 PMC7398139 32582958

[67] D. L. Bhatt , Cardiovascular risk reduction with icosapent ethyl for hypertriglyceridemia. N. Engl. J. Med. **380**, 11–22 (2019). 30415628 10.1056/NEJMoa1812792

[68] A. Majithia , Benefits of icosapent ethyl across the range of kidney function in patients with established cardiovascular disease or diabetes: REDUCE-IT RENAL. Circulation **144**, 1750–1759 (2021). 34706555 10.1161/CIRCULATIONAHA.121.055560 PMC8614567

[69] W. S. Harris, Understanding why REDUCE-IT was positive - Mechanistic overview of eicosapentaenoic acid. Prog. Cardiovasc. Dis. **62**, 401–405 (2019). 31666183 10.1016/j.pcad.2019.10.008

[70] L. C. Wu , Correction of sickle cell disease by homologous recombination in embryonic stem cells. Blood **108**, 1183–1188 (2006). 16638928 10.1182/blood-2006-02-004812 PMC1895869

